# Establishing usable innovations

**DOI:** 10.3389/frhs.2025.1745148

**Published:** 2026-01-16

**Authors:** Dean L. Fixsen, Melissa K. Van Dyke, Karen A. Blase

**Affiliations:** Active Implementation Research Network, Inc., Albuquerque, NM, United States

**Keywords:** evidence-based program, fidelity, scaling, usability testing, usable innovation

## Abstract

The persistence of the science to service gap is evidence that evidence is not enough when defining evidence-based programs. Innovations must be developed with attention to the internal and external validity of the innovations themselves so that innovations can be replicated and scaled. This paper outlines the requirements for establishing an innovation, recommends standards for a usable innovation, and describes the usability testing processes to meet those requirements. Usability testing is a systematic process to efficiently and effectively determine the essential components and to develop a fidelity measure for an innovation. Usability testing is the foundation for research to establish the internal validity (“the basic minimum without which any experiment is uninterpretable”) and external validity (“asks the question of generalizability”) of the innovation itself. Once the essential components of a usable innovation are defined, measured, and linked with outcomes, implementation and scaling of usable innovations with fidelity can narrow the science to service gap.

## Background

The science to service gap (1, 2) has persisted with few examples of using and scaling evidence-based programs in practice (3–5), and little evidence of meaningful benefits to populations (6, 7). Efforts to close the science to service gap have focused on developing evidence-based programs (8–10). In the absence of meaningful uptake of evidence-based programs (11), calls were made to improve the scientific rigor of outcome studies so that evidence would be more convincing and lead to greater use (12–15). The persistence of the science to service gap is evidence that defining evidence and setting standards to improve the quality of evidence are not enough.

A second line of inquiry focused on defining the innovation (practice, program, intervention), the independent variable in research to evaluate outcomes (science) and the dependent variable for research to evaluate the outcomes of implementation processes to put innovations into effect (service) (16). The definition of an innovation became the focus of debates regarding the effectiveness of therapy: what was it that was judged to be effective or ineffective (17–20)? As research on innovations progressed, the defining features of an innovation and the measurement of those features came to be viewed as inextricably entwined. The essential components had to be known to be measured and had to be measured to know that the essential components were present (21–23).

Systematic reviews concerning the use of innovations with fidelity in outcome studies (24–27) noted that the prerequisite to measuring fidelity is the specification of the essential components to be delivered. Suggestions to tailor or adapt evidence-based programs also center on the essential components. Damschroder, Aron (28), Kilbourne, Neumann (29), Szulanski (30), and others relate adaptability and refinement of innovations to the clarity of the essential components, “the essential and indispensable elements” of the innovation itself. Greenhalgh, Robert (31) called the essential components the “hard core,” and the adaptable elements the “soft periphery.” As Teague, Bond (32) stated, “without detailed descriptions of interventions, replication is difficult; without reliable measurement of interventions, conclusions about presence or absence of effects are questionable.”

With attention drawn to the essential components of innovations, Campbell, Fitzpatrick (33) cautioned, “There are specific difficulties in defining, developing, documenting, and reproducing complex interventions that are subject to more variation than a drug.” The need to specify the essential components and the difficulties in determining those components have been persistent issues in the field (29, 34–38). Damschroder, Aron (28) state, “A component analysis can be performed to identify the core vs. adaptable periphery components, but often the distinction is one that can only be discerned through trial and error over time as the intervention is disseminated more widely and adapted for a variety of contexts.”

A third line of inquiry resulted from attempts to use evidence-based programs in practice. As often happens in science (39), solving one set of problems reveals the next, previously unknown, set of problems. The science to service gap was amplified when evidence-based programs were available but not used in practice (1, 2, 40–42). And, when they were used, they often were not used as intended and did not produce expected outcomes (24–27). In addition, the planned processes to put something into effect that define implementation (28, 31, 43, 44) are themselves interaction-based innovations and, therefore, are included in the descriptions of innovations in this paper. Implementation science has its own science to service gap (45–48). Thus, there is a need to get innovation science right before attempting to use science in service.

This paper addresses how to establish the essential components of innovations so that they can be replicated and scaled in attempts to use science in service.

## Establishing innovations

The National Institutes of Health (NIH) in the US developed a Stage Model for behavioral intervention development (49). The stages are: Stage 0, basic science, Stage I, intervention generation, refinement, modification, and adaptation and pilot testing; Stage II, traditional efficacy testing; Stage III, efficacy testing with real-world providers; Stage IV, effectiveness research; and Stage V, dissemination and implementation research. In every stage, researchers are encouraged to examine the mechanisms of behavior change. The goal is to produce highly effective and scalable behavioral interventions that improve health and well-being. The NIH stages rely on traditional research designs and statistical analyses to move from one stage to another with little attention to the innovation itself.

In 2008 the Medical Research Council in the UK provided an overview of complex interventions that contain several interacting components and noted the difficulties encountered when attempting to evaluate them in practice (50). In 2019 the Medical Research Council offered guidelines for developing innovations (51) that include recommendations to use iterative cycles to develop a version of the intervention. These recommendations, and others (52, 53), do not include methods for conducting iterative cycles and no criteria were established for stopping the intensive development phase and moving on to using the innovation in practice. In this paper, an approach for establishing innovations is described and operationalized and includes explicit criteria for “moving on.”

The approach to establishing innovations is based on a different way of thinking about how to close the science to service gap to achieve significant benefits. To inform this way of thinking, definitions of key terms used in this paper are provided here in an effort to develop common concepts, common language, and common measures to advance a science of implementation. To *establish* something is to “bring it into existence, to make it firm and stable, to put it on a firm basis, to put it beyond doubt” (https://www.merriam-webster.com). *Original research* establishes the outlines of an innovation and its outcomes, an indication that subsequent research might be worthwhile. *Subsequent research*, based on usability testing, establishes a usable innovation that is able to be replicated and scaled, an indication that socially significant benefits might be achieved in practice. *Implementation* is an applied science where implementation is defined as the use of planned processes for putting something into effect. An *innovation* refers to an interaction-based practice or program that specifies the nature of certain exchanges between and among individuals or groups so that recipients will benefit (16).

The *essential components* that define the innovation are the features that must be present to say that an innovation exists in a given application, “the basic minimum without which any experiment is uninterpretable” (28, 54), and that must be present to achieve intended outcomes when replicating or scaling an innovation (55). *Evidence* is “facts, information, etc. that give reasons for believing that something is true or present” (https://dictionary.cambridge.org). *Internal validity* is the extent to which the essential components of an innovation are identified, measured, and linked to outcomes. *External validity and consequential validity* (the uses and benefits of evidence) (16) are primary considerations and “our methods [are] tools, convenient only insofar as they help us get there” (56).

With respect to *causality*, the US National Institutes of Health (NIH) refer to three criteria: temporal precedence, meaning that the hypothesized cause happens before the measured effect; covariation of the cause and effect, meaning that there is an established relationship between the two variables regardless of causation; and a lack of plausible alternative explanations (i.e., counterfactuals, the likely outcomes if the intervention had not been present) (https://www.nlm.nih.gov/oet/ed/stats/02-400.html). NIH considers a correlation of ±0.70 to be a strong correlation. *Causal evidence* regarding an independent variable (an innovation) “is *sufficient* if its presence is associated with the presence of the outcome, and… is *necessary* if the outcome is present only if the [innovation] is present” [(57); emphasis added]. With respect to innovations, research is done to establish *sufficient evidence* of a functional relationship between an innovation and its outcomes.

Currently, original research methods are well defined and multiple websites are devoted to rating innovations based on an examination of the rigor of the evidence [e.g., (58); https://www.evidenceforessa.org/]. While rating rigor is useful, the science to service gap remains, and scaling for population benefit is not common. Subsequent research is needed to establish innovations that are well defined, usable, assessable, and scalable. “Current research paradigms generally have not provided the answers needed … to produce research with more rapid clinical, public health, and policy impact” (4). As outlined above, a different way of thinking (59–61) is needed to guide subsequent research to establish usable innovations with attention to the internal and external validity of the innovations.

### Usability testing

Usability testing is an evidence-based method for identifying and operationalizing the essential components of interaction-based innovations. While the phrase “usability testing” is not common, the plan-do-study-act-cycle (PDSAC) logic and the use of small samples to develop functional relationships is recognizable in efforts to establish complex innovations (52, 62–67). For interaction-based innovations, the usability testing process continues until the essential components of an innovation are identified and operationalized (i.e., what to do and say), the essential components are assessed (i.e., a fidelity measure), and intended outcomes are achieved reliably (i.e., a strong correlation between the essential components and outcomes). While the logic of usability testing has been in use as innovations have been developed, the internal validity of innovations will be strengthened by purposefully using usability testing processes as intended.

Unlike trial and error, usability testing is a systematic *trial and learning* approach to “working out the bugs” in any complex program or system intended for general use (68–70). Usability testing originally was developed to test and improve the user interface with complex websites (e.g., merchandise marketing and sales) or computer hardware-software programs (e.g., video games). Once original research creates a prototype innovation, subsequent research is conducted with usability testing processes. In subsequent research, a usability testing team recruits a small number of participants (n∼5) to use the innovation. A testing team member may sit with a participant to observe the use of the innovation and ask questions about user decisions as they are made or ask the user to reflect on the content or style of a section of the innovation. Or the testing team may analyze data produced in the process of the participant using the innovation (e.g., time spent on a page, correct-incorrect use of specific features). Using information from the first participants, the innovation is revised to correct errors or to improve the user experience. Then the process is repeated with a new group of about 5 participants. Three to 5 cycles may be sufficient to detect and correct 80% or more of the errors eventually found in the original form of an innovation (71).

Based on PDSAC logic (72–75), in usability testing the Plan is to use the essential components of the innovation. Each participant then Does the plan. The research team Studies the results: did the individuals Do the Plan (fidelity) and to what extent were intended outcomes achieved? The research team Acts on the information by changing the innovation or the supports for users. In the next Cycle a new group of participants (n∼5) uses the hopefully improved essential components of the next version of the innovation (the new Plan). The Cycle is repeated until a successful outcome is achieved and the internal validity of the innovation is established.

In usability testing, the evolving fidelity assessment (Do the Plan; how do you know?) is used as the standard so that factors that negatively impact achieving the fidelity standard in each iteration can be detected and corrected without compromising the function—if this, then that [(76); chapter 13]. Fidelity is the “bug detector,” an indication that something is getting in the way of doing what is required to produce desired outcomes. As usability testing progresses, the presumed essential components are changed, deleted, and expanded. With each iteration, fidelity-outcome evidence is generated and “the pool of effective methods expands to incorporate effective responses to what previously was unanticipated. The expanded methods then can benefit a greater proportion of the variations encountered in communities, service settings, and organizations” (16). As the essential components become clearer, the fidelity assessment (i.e., the measure of the essential components) becomes more focused on the essential components and more highly correlated with outcomes. The internal validity of an innovation is established when fidelity of the use of the essential components correlates strongly (±0.70) with intended outcomes.

To establish external validity, Barker, Reid (67) advocate usability testing with increasing numbers of users to ensure exposure to an expanding range of real-life situations. Barker, Reid (67) provide examples where “the rate of expansion can be exponential (i.e., not linear) by a multiple of 5 … (e.g., 1–5–25–125–625, etc.).” At each level, external validity is strengthened as revised methods (Plan) are established to resolve newly exposed problems (Do, Study, Act) before moving to the next level (Cycle). Changes that do not improve usability and outcomes are discarded. Changes that improve usability and outcomes become part of the definition of an innovation as exposure to new problems invites new solutions so that the problems are solved and more beneficial outcomes are achieved. In this way, “the pool of effective methods” is expanded to include constructive responses to variations related to culture, race, gender, socio-economic conditions, geography, seasons, territorial conflicts, local contexts, and so on. Usability testing also can detect the limits of the use of the innovation, that is, the conditions under which the essential components do not produce the outcomes found under other conditions. Thus, the threshold for external validity of an innovation is the ability to replicate the usable innovation with fidelity in multiple real-life situations.

With usability testing as a guide for conducting subsequent research, functional cause and effect relationships are established for an innovation. Establishing cause and effect relationships is done with iterative small samples of users [referred to as small-n research (77)]. In the usability testing process, the sufficient conditions for achieving beneficial outcomes are established and the unnecessary and insufficient conditions are removed from the innovation (57, 78). The result is an innovation that is more efficient to use in practice while maximizing effectiveness.

An example regarding a new potential treatment for cancer is provided by Shadish et al. (79).

In the late 1990s, a team of researchers … reported that a new drug called Endostatin shrank tumors by limiting their blood supply (Folkman, 1996). Other respected researchers could not replicate the effect even when using drugs shipped to them from Folkman's lab. Scientists eventually replicated the results after they had traveled to Folkman's lab to learn how to properly manufacture, transport, store, and handle the drug and how to inject it in the right location at the right depth and angle. One observer labeled these contingencies the “in-our-hands” phenomenon, meaning “even we don't know which details are important, so it might take you some time to work it out” (Rowe, 1999). Endostatin … was insufficient cause by itself, and its effectiveness required it to be embedded in a larger set of conditions that were not even fully understood by the original investigators.

As noted in this example, the full set of essential components can be identified and assessed in subsequent research with each small group attempting to use the innovation. The endostatin example points out that any condition pertaining to the use of an innovation may qualify as an essential component. If judgement is required “to inject it in the right location at the right depth and angle,” then judgement is an essential component of the usable innovation, and the “right location and right depth” are assessed by the fidelity measure.

Another example of usability testing is based on the experience of establishing the Teaching-Family Model (1967-present). The Teaching-Family Model is an early example of an evidence-based program. The research and development of the Teaching-Family Model have been described and summarized (80–85). It was cited as a “model program” by the American Psychological Association in its initial review of “evidence-based programs” (86), as one of three evidence-based residential programs in the Surgeon General's report (87), and as perhaps the best developed and researched residential treatment model (88) among those reviewed by the California Evidence Based Clearinghouse for Child Welfare. Subsequent meta-analyses have found the Teaching-Family Model to be one of three residential treatment programs that produce positive and cost-beneficial outcomes (89, 90).

In retrospect, usability testing began early in the innovation development process. The Teaching-Family Model began in 1967 at Achievement Place, a group home for adjudicated youths in Lawrence, Kansas. A Teaching-Family group home is staffed by Teaching-Parents (a married couple and their children) who live in the home and provide family-style care and treatment for up to seven adjudicated youths referred by the local juvenile court. After 3 years of intensive research to develop treatment methods, the first attempt to replicate the Teaching-Family group home program failed in 1971 (84). The failure led to a re-examination of the treatment program (usability testing). Repeated analyses of videotaped interactions in the prototype group home and the replication group home revealed an essential function in the prototype program that had not been identified previously. The essential function was named the *teaching interaction*, a set of skills for teaching a wide variety of appropriate alternative behaviors. The Teaching-Parents at Achievement Place were teaching the youths appropriate behavior so skillfully it had been overlooked until the contrasting experience was observed in the replication group home. Another previously unrecognized component was relationship development. Again, so skillfully done it was not “seen” until its absence was noted in the replication home. The Teaching-Parents at Achievement Place used high rates of descriptive praise, statements of care and concern, and advocacy for youths in a natural way that fit into the flow of interactions in daily life. Usability testing added these new treatment components to the definition of the Teaching-Family Model.

The developers recognized they had failed to prepare the Teaching-Parents in the replication group home to use the evidence-based program as intended. In response to the failure, the developers discarded the previous academic methods for preparing Teaching-Parents and established new skill-based training and coaching processes to develop a broader set of defined competencies for Teaching-Parent couples who had been carefully selected for their roles as practitioners (82, 91–93). As the essential components and practitioner skill sets became clear, a fidelity assessment was developed and put into use (81, 92). The Teaching-Parents who had staffed the failed replication reapplied and participated in the new competency development processes and met fidelity criteria for several years in a new group home.

In 1972 three other group homes began using the Teaching-Family Model and the new learning was applied. By the end of 1975, the developers had attempted to replicate the Teaching-Family Model in 64 group homes, learning and applying new lessons along the way (94, 95). During the eight years from 1967 to 1975 the essential components of the Teaching-Family Model treatment program and implementation processes had been established, revised, refined, and evaluated. Fixsen, Blase [(76); chapter 4] provide a detailed review of the usability testing that led to the development of the essential components of the Teaching-Family Model and the implementation processes to replicate the residential (group home) treatment program for adjudicated youths.

## Usable innovations

McGrew, Bond (96) outlined the requirements for an innovation: (1) define interventions conceptually, (2) identify critical ingredients, (3) operationally define critical ingredients, (4) establish agreed-upon criteria for use in practice, and (5) study systematic variations with continued use. These requirements are reflected in the four criteria for a *usable innovation* (76, 97) outlined here and described in the following sections.

1. Description of the underlying philosophy, values, and principles and the participant inclusion and exclusion criteria.
2. Description of the identified essential components.
3. Operational definitions of the essential components (what practitioners do, say).
4. Practical fidelity assessment of the essential components strongly correlated (≥0.70) with desired outcomes.

### Philosophy, values, principles

The first criterion for a usable innovation is a description of the underlying philosophy, values, and principles along with the inclusion and exclusion criteria for intended recipients. The philosophy, values, and principles that underlie the innovation provide guidance for all innovation-related decisions and evaluations. When all else fails, practitioners fall back on philosophy, values, and principles as they decide how to cope with atypical challenges. For example, the Teaching-Family Model philosophy emphasizes ecologically appropriate treatment (family, peers, school, community); values include providing care and treatment that is humane, effective, individualized, satisfactory to recipients and consumers, cost efficient, and replicable; and uses principles derived from applied behavior analysis concerning teaching appropriate alternative behavior, positive motivation, self-determination, and social validity (81, 83, 98). Even under stressful circumstances, when all else fails, practitioners are (at a minimum) humane and caring in their interactions with youths and others.

The description of the innovation also states the inclusion and exclusion criteria that define the population for which the innovation is intended. The criteria define who is most likely to benefit when the innovation is used as intended, and who is not likely to benefit. For example, Multisystemic Therapy (MST) is an evidence-based program that provides homebased treatment services for youths and their families (99). MST includes youths who are (a) serious juvenile offenders (b) at imminent risk of placement in residential care (incarceration) and (c) living with at least one parent or adult caregiver. MST excludes (a) youths who are actively psychotic or in need of crisis psychiatric hospitalization or stabilization and (b) youths who have a sole diagnosis of autism or pervasive developmental disorder (100).

### Essential components

The second criterion for a usable innovation is a description of the identified essential components. For the purpose of developing a usable innovation, essential components are the features that must be present to say that an innovation exists in a given application. Essential components distinguish one innovation from another, and from standard practice. For example, Assertive Community Treatment (ACT) is an evidence-based program that provides intensive community-based treatment for individuals with severe mental illness (101–103). McHugo, Drake (104) describe the nine essential components of ACT as community locus, assertive engagement, high intensity, small caseload, continuous responsibility, staff continuity, team approach, multidisciplinary staff, and close work with support systems. In another example, the essential components of MST (99, 100) include finding the fit, positive and strength focused, increasing responsibility, present-focused, action-oriented & well-defined, targeting sequences, developmentally appropriate, and continuous effort (http://www.mstservices.com).

### Operational definitions

The third criterion for a usable innovation is operational definitions of the essential components. Operational definitions turn concepts into actions (what practitioners do and say). Operational definitions are the basis for developing high levels of competence and reducing variability related to ineffective, incomplete, or inconsistent uses of innovations across practitioners. Operational definitions specify the activities minimally required to carry out each essential component of an innovation, that is, when, where, how, and what to do and say when interacting with others to carry out each essential component of an innovation. Operational definitions also may specify proscribed activities that interfere with the use of an essential component and must be avoided. An example of an operational definition is provided in the Appendix.

Operational definitions are advocated by Aarons, Hurlburt (105) who point to the need for a “high degree of procedural specificity in work activities.” And they are a good fit with the recommendations of Chinman, Imm (106) and Greenhalgh, Robert (31) to develop “innovation configurations” as described by Hall and Hord (107). Innovation configurations (practice profiles) provide specific examples of expected behavior related to each essential component of an innovation.

### Fidelity assessment

The fourth criterion for a usable innovation is practical fidelity assessment where fidelity is strongly correlated (±0.70) with desired outcomes. A fidelity assessment is the product of usability testing where Doing a plan is assessed in the Study component and the assessment is related to outcomes. At the point where fidelity scores and outcome scores correlate at ±.70, then the fidelity assessment is sufficient to be used when attempting to replicate or scale the innovation. A fidelity assessment relates to the innovation philosophy, values, and principles; essential functions; and core activities that operationalize each essential function (108). The direct link between essential components and indicators of the presence and strength of those components means that a fidelity assessment always is specific to an innovation. A fidelity assessment is practical and can be done repeatedly in the context of typical usage of the innovation.

Thus, a usable innovation always has a fidelity measure to detect the presence and strength of the well-defined essential components of an innovation as it is used in practice. A strong correlation between fidelity of the use of the innovation and outcomes means that innovations that are used as intended (with high fidelity) reliably produce intended outcomes. This also means innovations that are not being used as intended (with low fidelity) produce poor outcomes or (sometimes) harmful outcomes (109). Therefore, users can expect that an innovation will be effective when used as intended.

An example of establishing a correlation is provided by Bedlington, Braukmann (110) [later published as Bedlington et al. (111)] who assessed teaching interactions, counseling interactions, and general social interactions between Teaching-Parents and youths in 14 Teaching-Family group homes. The Teaching-Parents were at various stages of their competency development and ability to use the Teaching-Family Model. Data from direct observations in each home found, on average, teaching interactions occurred 20% of the time, social interactions 39% of the time, and counseling interactions 4% of the time. Youth delinquency outcomes were assessed using a standard measure. Teaching interactions correlated −0.86 with delinquency while social interaction and counseling interactions correlated −0.23 and −0.24 with delinquency. In addition, youth satisfaction with the fairness, helpfulness, effectiveness, pleasantness, and concern of their Teaching-Parents was assessed using standard individual youth interview protocols. Teaching interactions correlated 0.73 with youth satisfaction while social interaction and counseling interactions correlated 0.20 and −0.14 with youth satisfaction. Teaching interactions are an essential component of the Teaching-Family Model and are highly correlated (−0.86 and 0.73) with socially significant outcomes. In this example, teaching is operationally defined, the teaching interaction is well specified and measurable, and the essential component is highly correlated with desired outcomes (i.e., internal validity was established).

In addition to the correlation data, it is helpful to contrast the outcomes for the top and bottom quintiles for fidelity. For example, the Washington State Institute for Public Policy (112) collected fidelity and outcome data for 25 therapists using Functional Family Therapy (FFT). FFT is an evidence-based program for providing treatment for adjudicated youth and their families (113, 114). Overall, there was a −0.61 correlation between fidelity and recidivism outcomes (number of youths who committed another felony offense during or after treatment). The quintile analysis found that youths in families treated by high fidelity (top 20%) FFT therapists had 8% recidivism and youths in families treated by low fidelity (bottom 20%) FFT therapists had 34% recidivism. Recidivism for youths in the control group was 22%. Quintile data provide meaningful information for potential users of a usable innovation and add credence to the causal inference.

The necessity of knowing the essential components of innovations underscores the value of fidelity assessments in research when attempting to develop an innovation (science) and when attempting to use innovations to benefit others (service) (108).

### Morals, ethics, judgement

Morals, ethics, and judgement may be defined as essential components of an innovation. Morals (what is right or wrong) can be assessed by asking recipients or others (e.g., family and community members) about the practitioners' “appropriate” or “humane” or “respectful” behavior and recipient outcomes. Ethics (what is good or bad) might relate to assessing behavior related to professional guidelines pertaining to effective and individualized care. Judgement (wisdom; good sense) might be assessed by observing when, where, how, and with whom practitioners interact, and doing and saying the right thing at the right time with the right person. Moral, ethical, and judgement issues likely will arise during usability testing as repeated iterations and scaling expose the developers to a wider range of people and circumstances. For direct observation and rating of morals, ethics, and judgement, the fidelity assessors need to be prepared to make judgements about judgement – what it is and how to observe it and how to rate it.

For example, fidelity assessments for the Teaching-Family Model include items that assess clinical judgement when using the essential components of teaching. In the homebased application of Teaching-Family treatment, one item asks the fidelity assessors to rate, “How satisfied are you that the Family Specialist [the practitioner] perceives and responds to teaching opportunities throughout the visit (effective praise, intervening directly when needed, attending to and noticing ongoing behavior)?” For fidelity assessors, “satisfaction” is defined and calibrated during the development of assessors' observation and rating skills, and routinely checked thereafter by calculating interobserver agreement between the two assessors who conduct an in-home visit (76, 115). During the development of assessors and periodically thereafter, the accuracy and quality of the comments accompanying a satisfaction rating also are the subject of discussion and agreement.

In another example, being positive and strength focused is an essential component of MST (116). MST asks family members to rate how the therapist behaved during a home visit, including questions related to judgement: “The therapist tried to understand how my family's problems all fit together.” and “Family members and the therapist agreed upon the goals of the session.” With these questions, it is up to the family to define “understand” and “agreed upon” in their own terms and from their own perspectives.

Once the criteria for a usable innovation are met (i.e., the explicit criteria for “moving on”), the intensive development phase can end, and the research group can move on to using the innovation to advance science and to engage in scaling.

## Usable innovations in practice

Meeting the four criteria for a usable innovation is preparation for being able to replicate and scale an innovation so that recipients can benefit more broadly. Usability testing takes extra time and effort up front, but developing a usable innovation saves time and effort and improves success when attempting to use implementation processes to put an innovation into practice.

For implementation and scaling, the essential components of a usable innovation are the foundation for each level of use. The essential components are known and produce intended outcomes, and it is that relationship that produces intended outcomes at each level of use – practitioner, organization, and system. Thus, the fidelity assessment can be relied on as the measure of successfully using the innovation and as a predictor of outcomes.

Logically, a practitioner uses an innovation so that each recipient can benefit from the essential components as the practitioner and recipient interact. In organizations, many practitioners use an innovation with fidelity and in systems many practitioners in many organizations use an innovation with fidelity. At each level, desired outcomes depend on high fidelity use of the essential components by each practitioner. The essential components do not change and the fidelity assessment does not change, but the number of practitioners whose fidelity is assessed increases at each level from one user to many thousands of users employed in organizations and enabled by systems (55, 117, 118).

As the use of an innovation moves from one user to many thousands, practitioner change, organization change, and system change are based on using the essential components of an innovation so that desired (improved) outcomes can be realized now and in the future. At the practice level, fidelity is the test of the practitioners' use of an innovation so that intended outcomes can be achieved. McIntosh, Mercer (119) studied 26,655 fidelity assessments conducted annually over 5 years in 5,331 schools located in 1,420 school districts in 37 states and found patterns in the fidelity assessments across years (sustainers, slow starters, late abandoners, rapid abandoners). Tommeraas and Ogden (117) documented the consistent high fidelity use of an evidence-based program with good outcomes for 10 years in Norway. And, Fixsen et al. (115) examined fidelity of the use of an innovation by all practitioners who were employed in a large residential treatment organization using the Teaching-Family Model as staff and leaders came and went over a period of 50 years. In these and other studies, fidelity provides a proximal indication that the essential components of the innovation are in use at an acceptable level by a practitioner.

At the organization level, fidelity is the test of implementation processes and administrative supports for the use of an innovation so that intended outcomes can be achieved. For example, organization change and sustained change in an organization was assessed using an “implementation quotient” measure (115). The measure combined data from the standard Teaching-Family Model fidelity assessment with data on staff tenure to document organization change. The combined data take into consideration practitioner competency development (fidelity) and availability of qualified staff as turnover occurs. The implementation quotient improved substantially (from 1.1 to 4.6 on a 5-point scale) during the eight years of intensive organization change (1975–1983) and was sustained (4.6) for 50 years (1975–2025). In another example, Glisson et al. (120) (a) randomly assigned adjudicated youth within each county in 14 rural Appalachian counties to a multisystemic therapy (MST) program or usual services and (b) randomly assigned counties to the ARC (Availability, Responsiveness, and Continuity) organizational intervention. Fidelity assessments ensured that each complex evidence-based innovation (MST and ARC) was being used and at sufficient strength to conduct a credible test. They found that the combination of evidence-based treatment (MST) and facilitative organization support (ARC) produced the best outcomes for adjudicated youths. In these examples, fidelity provides a strong indication that the essential components of the innovation are in use at an organization level.

At the system level, fidelity is the test of systemic conditions that enable many organizations to use implementation processes so that many more practitioners can use an innovation so that outcomes can be achieved on a meaningful scale. For example, to eradicate smallpox, in the 1960s surveillance teams were developed to identify outbreaks of smallpox and containment teams were developed to isolate and treat the infected and inoculate the exposed. These usable innovations were the result of usability testing conducted in Africa (121). For each team, fidelity was frequently assessed and promptly reported so that constant improvements could be made as the innovations were used in each country. At a national scale in India, hundreds of surveillance teams contacted 140,000 villages in one state in one month with fidelity assessed for each team. The last case of smallpox globally was found and treated in 1979. In another example, Ogden and colleagues (117, 122–127) describe the development of a system to establish, scale, and sustain fidelity and outcomes for evidence-based programs nationally in Norway. Ogden, Bjørnebekk (123) assessed the implementation processes that had been established for two innovations in the behavioral health system in Norway and Tommeraas and Ogden (117) reported the continuing high levels of fidelity and outcomes for 10 years. “Generations” of practitioners used the usable innovation with fidelity, and generations of children and families benefitted (126).

As these examples illustrate, with usable innovations the essential components are defined, present, and functional, and fidelity is the measure of their presence and strength at every level of use. This is not to say that other process and outcome measures are not needed as usable innovations are scaled to produce socially significant benefits. However, fidelity assessment always is the first measure to ensure the essential components-fidelity-outcomes link at the level of service delivery. Essential components are assessed with a fidelity measure and fidelity is highly correlated with outcomes. A usable innovation provides the information and confidence needed to implement and scale innovations so that populations can benefit.

## Discussion

The processes to establish usable innovations are outlined to advance science and service. Usability testing is described as a systematic process to efficiently and effectively determine the essential components and to develop a fidelity measure for an innovation. Usability testing is the foundation for research to establish the internal validity (“the basic minimum without which any experiment is uninterpretable”) and external validity (“asks the question of generalizability”) of the innovation itself. A usable innovation meets the requirements for an innovation as outlined by McGrew et al. (96).

A usable innovation is important for resolving some of the persistent issues for using science in practice to benefit people and society. A usable innovation, by definition, has essential components that are known, are measured by a fidelity assessment, and are strongly correlated with beneficial outcomes. As an independent variable, a usable innovation provides a clear and replicable link to outcomes. With the strong link to outcomes established as a result of the usability testing process (108), usable innovations define the essential components that must be present in the “hard core,” leaving the non-essential components in the “soft periphery” that can be adapted (28, 31). High fidelity use of the essential components of a usable innovation is not an option, it is a requirement for producing intended benefits. “Tailoring” and “adaptation” already have occurred as part of the usability testing process and the essential components are the result.

In group designs that require larger numbers of participants, the independent variable (i.e., the usable innovation) is well defined, fidelity assesses its presence and strength, and a major source of error variance is reduced or accounted for. As a dependent variable in studies of the use of innovations in practice, the fidelity assessment for a usable innovation provides a clear measure of the presence and strength of the innovation in use. Thus, if an implementation process (the independent variable) results in high fidelity use of an innovation, then there is evidence that the implementation process is effective. Science can advance more rapidly and confidently once usable innovations are established.

For decades it has been stated that the essential components of innovations are difficult to determine. Essential components do require time and attention beyond the original research to study an innovation and its outcomes. In recounting the remarkable history of invention (e.g., the transistor) and application (science to service in communication systems) at Bell Telephone Laboratories since the 1920s, Gertner (128) estimated that “pursuing an idea takes … fourteen times as much effort as having it” (p. 348). When “pursuing” an innovation, usability testing clarifies the essential components as they are established, tested, revised, re-established, and retested until fidelity is high and intended outcomes are achieved. The essential components of an innovation then can be relied on to discriminate good examples from poor examples, and one innovation from another.

Establishing usable innovations requires changing views of innovations and how they are developed. As Kurt Lewin (129, 130) noted with respect to organization change, the first step in the process (“unfreeze”) involves letting go of certain restricting attitudes so that preconceptions are altered and new ways of thinking (“change”) can lead to improved results. The new ways of thinking outlined in this paper need to become the standard way (“refreeze”) to establish usable innovations and contribute to the science of innovations. In this new way of thinking, the websites rating evidence-based innovations based on the rigor of the original research may point to innovations that are worth developing. Before attempting to use them with an expectation of improved benefits for others, the next step is subsequent research based on usability testing so that effective innovations can become usable innovations that can be replicated and scaled to benefit populations.

The usability testing process for developing usable innovations has direct relevance to the NIH Stage Model and the UK Medical Research Council guidelines. The usability testing process operationalizes the recommendations of the Medical Research Council to use iterative cycles to develop the intervention. The usability testing process also achieves many of the goals NIH envisioned for Stage I (intervention generation, refinement, modification, and adaptation and pilot testing), Stage II (efficacy testing), and Stage III (efficacy testing with real-world providers). Only when the usable innovation criteria are met is the research group ready for Stage IV (effectiveness research) and Stage V (dissemination and implementation research). Without high fidelity use of the essential components there is little chance of achieving the goal of the Stage Model, that is, to produce highly effective and scalable behavioral interventions that improve health and well-being.

To put something into effect, one time or at scale, the something must be known and repeatable. The examples of usable innovations in practice cited in this paper provide evidence of what is possible. Armed with usable innovations and with fidelity of the use of those innovations as the goal, then scaling can achieve desirable outcomes for whole populations. With usable innovations, the science to service gap can become the science to service pathway.

## Data Availability

The original contributions presented in the study are included in the article/Supplementary Material, further inquiries can be directed to the corresponding author.

## Appendix

For the Teaching-Family Model, the Teaching-Parents (practitioners) use the teaching interaction to teach youths (and others) appropriate alternative behavior. Teaching is an essential component of the Teaching-Family Model along with relationship development, motivation systems, self-determination, and counseling. With teaching appropriate alternative behavior as an essential component of the Teaching-Family Model (81, 91), a teaching interaction is operationally defined as:

Qualitative Components

○ Use a calm, caring speaking voice
○ Be enthusiastic and positive when praising
○ Be calm and matter of fact when offering corrective feedback
○ Stay in close proximity
○ Use polite and pleasant requests (please …, would you …)

Behavior Components

- Initial positive statement
  ○ Begin with a statement of praise, empathy, concern
  ○ Set a positive tone for the interaction
- Name the skill (use a concept label)
  ○ The focus for the interaction
- Describe the inappropriate behavior (reactive teaching only)
  ○ Give a specific description (a factual replay of what happened or was omitted)
  ○ Demonstrate what cannot be described (facial expressions, gestures)
  ○ Do not blame or mock the youth (be non-judgmental)
- Describe the negative consequence (reactive teaching only)
  ○ Mention loss of access to privileges, points, checkmarks
  ○ State positive correction (ability to earn back half the loss by promptly practicing appropriate alternative behavior)
- Describe the appropriate behavior
  ○ Restate the skill label
  ○ Provide a specific description (examples of what to do and say)
  ○ Demonstrate what cannot be described (voice tone, facial expressions)
- Give a rationale
  ○ Make a brief, personal, believable statement about the likely consequences in environments important to the young person
  ○ Point out the short-term natural benefits of using the skill or harms of not using the skill
  ○ Link skill label, behaviors, and outcomes
- Request acknowledgement
  ○ Check for understanding throughout the interaction
- Practice
  ○ State skill label
  ○ Describe/demonstrate appropriate behavior components
  ○ Set up the practice “scene”
  ○ Act out the scene (role play)
- Practice feedback
  ○ Give effective praise for parts of practice that went well
  ○ Provide corrective feedback for parts that need improvement
  ○ Repracticing to criterion until youth is comfortable with new skill
  ○ Reinforce positive consequences (positive correction: earn back half of any loss)
  ○ Restate benefits of using the skill in the future
- General praise
  ○ Give praise for engaging in the interaction (cooperation)
  ○ Encourage the effort to learn

## References

[1] Institute of Medicine. Crossing the Quality Chasm: A New Health System for the 21st Century. Washington, D.C.: National Academy Press (2001). 25057539

[2] Weisz JR Donenberg GR Han SS Weiss B. Bridging the gap between laboratory and clinic in child and adolescent psychotherapy. J Consult Clin Psychol. (1995) 63:688–701. 10.1037/0022-006X.63.5.688 7593861

[3] Perl HI. Addicted to discovery: does the quest for new knowledge hinder practice improvement? Addict Behav. (2011) 36(6):590–6. 10.1016/j.addbeh.2011.01.027 21349648

[4] Kessler RC Glasgow RE. A proposal to speed translation of healthcare research into practice: dramatic change is needed. Am J Prev Med. (2011) 40(6):637–44. 10.1016/j.amepre.2011.02.023 21565657

[5] United Nations. The Sustainable Development Goals Report. New York: United Nations Publications (2023).

[6] Kruk ME Gage AD Joseph NT Danaei G García-Saisó S Salomon PJA. Mortality due to low-quality health systems in the universal health coverage era: a systematic analysis of amenable deaths in 137 countries. Lancet. (2018) 392(10160):2203–12. 10.1016/S0140-6736(18)31668-4 30195398 PMC6238021

[7] National Center for Education Statistics. The Nation s Report Card: Trends in Academic Progress 2012. Washington, DC: Institute of Education Sciences, U.S. Department of Education: Institute of Education Sciences, U.S. Department of Education (2013). Available online at: http://nces.ed.gov/nationsreportcard/subject/publications/main2012/pdf/2013456.pdf (Contract No.: NCES 2013 456).

[8] Hoagwood K Burns BJ Kiser L Ringeisen H Schoenwald SK. Evidence-based practice in child and adolescent mental health services. Psychiatr Serv. (2001) 52(9):1179–89. 10.1176/appi.ps.52.9.1179 11533391

[9] Roberts MC, editor. Model Programs in Child and Family Mental Health. Mahwah, NJ: Lawrence Erlbaum Associates (1996).

[10] Sackett DL Rosenberg WMC Gray JAM Haynes RB Richardson WS. Evidence-Based medicine: what it is and what it isn't. Br Med J. (1996) 312:71–2. 10.1136/bmj.312.7023.71 8555924 PMC2349778

[11] Balas EA Boren SA. Managing clinical knowledge for health care improvement. Yearb Med Inform. (2000) 1:65–70. 27699347

[12] Carroll KM Rounsaville BJ. Bridging the gap: a hybrid model to link efficacy and effectiveness research in substance abuse treatment. Psychiatr Serv. (2003) 54(3):333–9. 10.1176/appi.ps.54.3.333 12610240 PMC3650626

[13] Tunis SR Stryer DB Clancy CM. Practical clinical trials: increasing the value of clinical research for decision making in clinical and health policy. JAMA. (2003) 290(12):1624–32. 10.1001/jama.290.12.1624 14506122

[14] Jenicek M Stachenko S. Evidence-based public health, community medicine, preventive care. Med Sci Monit. (2003) 9(2):Sr1–7. 12601308

[15] Altman DG Schulz KF Moher D Egger M Davidoff F Elbourne D The revised CONSORT statement for reporting randomized trials: explanation and elaboration. Ann Intern Med. (2001) 134(8):663–94. 10.7326/0003-4819-134-8-200104170-00012 11304107

[16] Fixsen DL Van Dyke MK Blase KA. Is implementation science a science? Not yet. Front Public Health. (2024) 12:1–13. 10.3389/fpubh.2024.1454268 PMC11521924 39478746

[17] Eysenck H. The effects of psychotherapy: an evaluation. J Consult Psychol. (1952) 16:319–24. 10.1037/h0063633 13000035

[18] Malan DH. The outcome problem in psychotherapy research: a historical review. Arch Gen Psychiatry. (1973) 29(6):719–29. 10.1001/archpsyc.1973.04200060005001 4584600

[19] Fiske DW Hunt HF Luborsky L Orne MT Parloff MB Reiser MF Planning of research on effectiveness of psychotherapy. Arch Gen Psychiatry. (1970) 22(1):22–32. 10.1001/archpsyc.1970.01740250024004 5409518

[20] Epstein NB Vlok LA. Research on the results of psychotherapy: a summary of evidence. Am J Psychiatry. (1981) 138(8):1027. 10.1176/ajp.138.8.1027 7258377

[21] Yeaton WH Sechrest L. Critical dimensions in the choice and maintenance of successful treatments: strength, integrity, and effectiveness. J Consult Clin Psychol. (1981) 49:156–67. 10.1037/0022-006X.49.2.156 7217482

[22] Dobson L Cook T. Avoiding type III error in program evaluation: results from a field experiment. Eval Program Plann. (1980) 3:269–76. 10.1016/0149-7189(80)90042-7

[23] Scheirer MA Rezmovic EL. Measuring the degree of program implementation:a methodological review. Eval Rev. (1983) 7(5):599–633. 10.1177/0193841X8300700502

[24] Moncher FJ Prinz RJ. Treatment fidelity in outcome studies. Clin Psychol Rev. (1991) 11:247–66. 10.1016/0272-7358(91)90103-2

[25] Gresham FM Gansle KA Noell GH. Treatment integrity in applied behavior analysis with children. J Appl Behav Anal. (1993) 26(2):257–63. 10.1901/jaba.1993.26-257 8331022 PMC1297745

[26] Naleppa MJ Cagle JG. Treatment fidelity in social work intervention research: a review of published studies. Res Soc Work Pract. (2010) 20(6):674–81. 10.1177/1049731509352088

[27] Durlak JA DuPre EP. Implementation matters: a review of research on the influence of implementation on program outcomes and the factors affecting implementation. Am J Community Psychol. (2008) 41:327–50. 10.1007/s10464-008-9165-0 18322790

[28] Damschroder LJ Aron DC Keith RE Kirsh SR Alexander JA Lowery JC. Fostering implementation of health services research findings into practice: a consolidated framework for advancing implementation science. Implement Sci. (2009) 4(50):1–15. 10.1186/1748-5908-4-50 19664226 PMC2736161

[29] Kilbourne AM Neumann MS Pincus HA Bauer MS Stall R. Implementing evidence-based interventions in health care: application of the replicating effective programs framework. Implement Sci. (2007) 2:42. 10.1186/1748-5908-2-42 18067681 PMC2248206

[30] Szulanski G. Exploring internal stickiness: impediments to the transfer of best practice within the firm. Strategic Manag J. (1996) 17(Special Issue):27–43. 10.1002/smj.4250171105

[31] Greenhalgh T Robert G MacFarlane F Bate P Kyriakidou O. Diffusion of innovations in service organizations: systematic review and recommendations. Milbank Q. (2004) 82(4):581–629. 10.1111/j.0887-378X.2004.00325.x 15595944 PMC2690184

[32] Teague GB Bond GR Drake RE. Program fidelity in assertive community treatment: development and use of a measure. Am J Orthopsychiatry. (1998) 68(2):216–32. 10.1037/h0080331 9589760

[33] Campbell M Fitzpatrick R Haines A Kinmonth AL Sandercock P Spiegelhalter D Framework for design and evaluation of complex interventions to improve health. Br Med J. (2000) 321(7262):694–6. 10.1136/bmj.321.7262.694 10987780 PMC1118564

[34] Mowbray CT Holter MC Teague GB Bybee D. Fidelity criteria: development, measurement, and validation. Am J Eval. (2003) 24(3):315–40. 10.1177/109821400302400303

[35] Michie S Fixsen D Grimshaw J Eccles M. Specifying and reporting complex behaviour change interventions: the need for a scientific method. Implement Sci. (2009) 4(1):40. 10.1186/1748-5908-4-40 19607700 PMC2717906

[36] Carroll C Patterson M Wood S Booth A Rick J Balain S. A conceptual framework for implementation fidelity. Implement Sci. (2007) 2(1):40. 10.1186/1748-5908-2-40 18053122 PMC2213686

[37] Kelly JA Heckman TG Stevenson LY Williams PN Hays RB Leonard NR Transfer of research-based HIV prevention interventions to community service providers: fidelity and adaptation. AIDS Educ Prev. (2000) 12(5):87–98. 11063072

[38] Tolin DF Grasso D Boness CL Beck JG Keane TM Leichsenring F A proposed definition of psychological treatment and its relation to empirically supported treatments. Clin Psychol. (2025) 32(3):213–25.

[39] Wootton D. The Invention of Science: A New History of the Scientific Revolution. New York: Harper Collins (2015).

[40] Bero L Grilli R Grimshaw J Harvey E Oxman A Thomson M. Closing the gap between research and practice: an overview of systematic reviews of interventions to promote the implementation of research findings. The Cochrane effective practice and organization of care review group. Br Med J. (1998) 317:465–8. 10.1136/bmj.317.7156.465 9703533 PMC1113716

[41] Goodman RM. Bridging the gap in effective program implementation: from concept to application. J Community Psychol. (2000) 28(3):309–21. 10.1002/(SICI)1520-6629(200005)28:3<309::AID-JCOP6>3.0.CO;2-O

[42] Antman EM Lau J Kupelnick B Mosteller F Chalmers TC. A comparison of results of meta-analyses of randomized control trials and recommendations of clinical experts: treatments for myocardial infarction. JAMA. (1992) 268(2):240–8. 10.1001/jama.1992.03490020088036 1535110

[43] Fixsen DL Naoom SF Blase KA Friedman RM Wallace F. Implementation Research: A Synthesis of the Literature: National Implementation Research Network. Tampa, FL: University of South Florida (2005). p. iii–119.

[44] Rycroft-Malone J. The PARIHS framework: a framework for guiding the implementation of evidence-based practice. J Nurs Care Qual. (2004) 19(4):297–304. 10.1097/00001786-200410000-00002 15535533

[45] Westerlund A Nilsen P Sundberg L. Implementation of implementation science knowledge: the research-practice gap paradox. Worldviews Evid Based Nurs. (2019) 16(5):332–4. 10.1111/wvn.12403 31603625 PMC6899530

[46] Proctor EK Bunger AC Lengnick-Hall R Gerke DR Martin JK Phillips RJ Ten years of implementation outcomes research: a scoping review. Implement Sci. (2023) 18(1):31. 10.1186/s13012-023-01286-z 37491242 PMC10367273

[47] Beidas RS Dorsey S Lewis CC Lyon AR Powell BJ Purtle J Promises and pitfalls in implementation science from the perspective of US-based researchers: learning from a pre-mortem. Implement Sci. (2022) 17(1):55. 10.1186/s13012-022-01226-3 35964095 PMC9375077

[48] Rapport F Smith J Hutchinson K Clay-Williams R Churruca K Bierbaum M Too much theory and not enough practice? The challenge of implementation science application in healthcare practice. J Eval Clin Pract. (2022) 28(6):991–1002. 10.1111/jep.13600 34268832

[49] National Institutes of Health. NIH stage model for behavioral intervention development (2022).

[50] Craig P Dieppe P Macintyre S Michie S Nazareth I Petticrew M. Developing and evaluating complex interventions: the new medical research council guidance. Br Med J. (2008) 337:a1655. 10.1136/bmj.a1655 18824488 PMC2769032

[51] O"Cathain A Croot L Duncan E Rousseau N Sworn K Turner KM Guidance on how to develop complex interventions to improve health and healthcare. BMJ Open. (2019) 9(8):e029954. 10.1136/bmjopen-2019-029954 PMC6701588 31420394

[52] Epstein D Klerman JA. When is a program ready for rigorous impact evaluation? The role of a falsifiable logic model. Eval Rev. (2012) 36:375–401. 10.1177/0193841X12474275 23420580

[53] Spicer N Hamza YA Berhanu D Gautham M Schellenberg J Tadesse F ‘The development sector is a graveyard of pilot projects!’ six critical actions for externally funded implementers to foster scale-up of maternal and newborn health innovations in low and middle-income countries. Global Health. (2018) 14(1):74. 10.1186/s12992-018-0389-y 30053858 PMC6063024

[54] Campbell DT Stanley JC. Experimental and Quasi-Experimental Designs for Research. Chicago: Rand McNally & Company (1963).

[55] Fixsen DL Blase KA Fixsen AAM. Scaling effective innovations. Criminol Public Policy. (2017) 16(2):487–99. 10.1111/1745-9133.12288

[56] Galea S. An argument for a consequentialist epidemiology. Am J Epidemiol. (2013) 178(8):1185–91. 10.1093/aje/kwt172 24022890

[57] Rohlfing I Zuber CI. Check your truth conditions! clarifying the relationship between theories of causation and social science methods for causal inference. Sociol Methods Res. (2021) 50(4):1623–59. 10.1177/0049124119826156

[58] Elliott DS Mihalic S. Blueprints for Violence Prevention. Boulder, CO: University of Colorado, Institute of Behavioral Science, Center for the Study and Prevention of Violence (2004).

[59] Sterman JD. Learning from evidence in a complex world. Am J Public Health. (2006) 96(3):505–14. 10.2105/AJPH.2005.066043 16449579 PMC1470513

[60] Holtrop JS Scherer LD Matlock DD Glasgow RE Green LA. The importance of mental models in implementation science. Front Public Health. (2021) 9:2021. 10.3389/fpubh.2021.680316 PMC8290163 34295871

[61] Hawe P. Lessons from Complex interventions to improve health. Annu Rev Public Health. (2015) 36(2015):307–23. 10.1146/annurev-publhealth-031912-114421 25581153

[62] Akin BA Bryson SA Testa MF Blase KA McDonald T Melz H. Usability testing, initial implementation, and formative evaluation of an evidence-based intervention: lessons from a demonstration project to reduce long-term foster care. Eval Program Plann. (2013) 41(0):19–30. 10.1016/j.evalprogplan.2013.06.003 23892175

[63] Henton M Rabin B Gaglio B Nekhlyudov L Dearing J Bull S A1-3: small-scale implementation study of the cancer survival query system. Clin Med Res. (2014) 12(1-2):77. 10.3121/cmr.2014.1250.a1-3

[64] Hirschhorn LR Semraul K Kodkany B Churchill R Kapoor A Spector J Learning before leaping: integration of an adaptive study design process prior to initiation of BetterBirth, a large-scale randomized controlled trial in Uttar Pradesh, India. Implement Sci. (2015) 10(117):1–9. 10.1186/s13012-015-0309-y 26271331 PMC4536663

[65] Harrison MI Grantham S. Learning from implementation setbacks: identifying and responding to contextual challenges. Learn Health Syst. (2018) 2(4):e10068. 10.1002/lrh2.10068 31245592 PMC6508762

[66] Rolock N Ocasio K Webb J Fleary-Simmons D Cohen L Fong R. Implementation science and prevention in action: application in a post-permanency world. J Evid Inf Soc Work. (2018) 16(1):1–17. 10.1080/23761407.2018.1517068 30303462

[67] Barker PM Reid A Schall MW. A framework for scaling up health interventions: lessons from large-scale improvement initiatives in Africa. Implement Sci. (2015) 11(1):12. 10.1186/s13012-016-0374-x PMC4731989 26821910

[68] Rubin J. Handbook of Usability Testing: How to Plan, Design, and Conduct Effective Tests. New York: John Wiley & Sons (1994).

[69] Genov A. Iterative usability testing as continuous feedback: a control systems perspective. J Usability Stud. (2005) 1(1):18–27.

[70] Nielsen J. Usability for the masses. J Usability Stud. (2005) 1(1):2–3.

[71] Nielsen J. Why You Only Need to Test with 5 Users. Alertbox (2000). Available online at: https://www.nngroup.com/articles/why-you-only-need-to-test-with-5-users/ (Accessed April 22, 2007).

[72] Speroff T O’Connor GT. Study designs for PDSA quality improvement research. Qual Manag Health Care. (2004) 13(1):17–32. 10.1097/00019514-200401000-00002 14976904

[73] Taylor MJ McNicholas C Nicolay C Darzi A Bel D Reed JE. Systematic review of the application of the plan–do–study–act method to improve quality in healthcare. Br Med J Q Safety. (2014) 23(4):290–8. 10.1136/bmjqs-2013-001862 PMC3963536 24025320

[74] Shewhart WA. Statistical Method from the Viewpoint of Quality Control. Garden City, NY: Dover Publications (1939).

[75] Deming WE. Out of the Crisis. Cambridge, MA: MIT Press (1986).

[76] Fixsen DL Blase KA Van Dyke MK. Implementation Practice and Science. 1st ed. Chapel Hill, NC: Active Implementation Research Network, Inc. (2019). p. 378.

[77] Mahoney J. Toward a unified theory of causality. Comp Polit Stud. (2008) 41(4-5):412–36. 10.1177/0010414007313115

[78] Mackie JL. Causes and conditions. Am Philos Q. (1965) 2(4):245–64.

[79] Shadish WR Cook TD Campbell DT. Experimental and Quasi-Experimental Designs for Generalized Causal Inference. New York: Houghton Mifflin Company (2002).

[80] Phillips EL Phillips EA Fixsen DL Wolf MM. Achievement place: modification of behaviors of Pre-delinquent boys within a token economy. J Appl Behav Anal. (1971) 4(1):45–59. 10.1901/jaba.1971.4-45 16795279 PMC1310667

[81] Phillips EL Phillips EA Fixsen DL Wolf MM. The Teaching-Family Handbook. 2nd ed. Lawrence, KS: University Press of Kansas (1974).

[82] Blase KA Fixsen DL Phillips EL. Residential treatment for troubled children: developing service delivery systems. In: Paine SC Bellamy GT Wilcox B, editors. Human Services That Work: From Innovation to Standard Practice. Baltimore, MD: Paul H. Brookes Publishing (1984). p. 149–65.

[83] Wolf MM Kirigin KA Fixsen DL Blase KA Braukmann CJ. The teaching-family model: a case study in data-based program development and refinement (and dragon wrestling). J Organ Behav Manage. (1995) 15:11–68. 10.1300/J075v15n01_04

[84] Fixsen DL Blase KA. The teaching-family model: the first 50 years. Persp Behav Sci. (2019) 42(2):189–211. 10.1007/s40614-018-0168-3 PMC6701480 31976429

[85] Phillips EL. Achievement place: token reinforcement procedures in a home-style rehabilitation setting for “pre-delinquent” boys. J Appl Behav Anal. (1968) 1:213–23. 10.1901/jaba.1968.1-213 16795179 PMC1311003

[86] Roberts MC Hinton-Nelson M. Models for service delivery in child and family mental health. In: Roberts MC, editor. Model Programs in Child and Family Mental Health. Mahwah, NJ: Lawrence Erlbaum Associates (1996). p. 1–21.

[87] U.S. Department of Health and Human Services. Mental Health: A Report of the Surgeon General. Rockville, MD: U.S. Department of Health and Human Services: Rockville, MD: U.S. Department of Health and Human Services (1999).

[88] James S. What works in group care?—a structured review of treatment models for group homes and residential care. Child Youth Serv Rev. (2011) 33(2):308–21. 10.1016/j.childyouth.2010.09.014 22468014 PMC3314708

[89] Lipsey MW Wilson DB. Effective intervention for serious juvenile offenders: synthesis of research. In: Loeber R Farrington DP, editors. Serious and Violent Juvenile Offenders: Risk Factors and Successful Interventions. Thousand Oaks, CA: Sage Publications, Inc. (1998). p. 1–7.

[90] Washington State Institute for Public Policy. Benefit-cost analysis: Juvenile justice (2016). Available online at: http://www.wsipp.wa.gov/benefitcost (Accessed December 15, 2017).

[91] Blase KA Maloney DM Timbers GD. Teaching-Parent Training Manual. Morganton, NC: Bringing It All Back Home Study Center (1974).

[92] Braukmann CJ Fixsen DL Kirigin KA Phillips EA Phillips EL Wolf MM. Achievement place: the training and certification of teaching-parents. In: Wood WS, editor. Issues in Evaluating Behavior Modification. Champaign, IL: Research Press (1975). p. 131–52.

[93] Braukmann CJ Kirigin-Ramp KA Tigner DM Wolf MM. The teaching family approach to training group home parents: training procedures, validation research, and outcome findings. In: Dangle R Polster R, editors. Behavioral Parent Training: Issues in Research and Practice. New York: Guilford Press (1984). p. 144–61.

[94] Fixsen DL Blase KA. Creating new realities: program development and dissemination. J Appl Behav Anal. (1993) 26:597–615. 10.1901/jaba.1993.26-597 8307838 PMC1297899

[95] Fixsen DL Blasé KA Timbers GD Wolf MM. In search of program implementation: 792 replications of the teaching-family model. Behav Anal Today. (2007) 8(1):96–110. 10.1037/h0100104

[96] McGrew JH Bond GR Dietzen L Salyers MP. Measuring the fidelity of implementation of a mental health program model. J Consult Clin Psychol. (1994) 62(4):670–8. 10.1037/0022-006X.62.4.670 7962870

[97] Blase KA Fixsen DL. Core Intervention Components: Identifying and Operationalizing What Makes Programs Work. Washington, DC: Office of the Assistant Secretary for Planning and Evaluation, Office of Human Services Policy, U.S. Department of Health and Human Services (2013).

[98] Wolf MM. Social validity: the case for subjective measurement or how applied analysis is finding its heart. J Appl Behav Anal. (1978) 11:203–14. 10.1901/jaba.1978.11-203 16795590 PMC1311293

[99] Henggeler SW Pickrel SG Brondino MJ. Multisystemic treatment of substance-abusing and -dependent delinquents: outcomes, treatment fidelity, and transportability. Ment Health Serv Res. (1999) 1(3):171–84. 10.1023/A:1022373813261 11258740

[100] Schoenwald SK Brown TL Henggeler SW. Inside multisystemic therapy: therapist, supervisory, and program practices. J Emot Behav Disord. (2000) 8(2):113–27. 10.1177/106342660000800207

[101] Stein LI Test MA. Alternatives to Mental Hospital Treatment. New York: Plenum Press (1978).

[102] Bond GR Salyers MP. Prediction of outcome from the dartmouth assertive community treatment fidelity scale. CNS Spectr. (2004) 9(12):937–42. 10.1017/S1092852900009792 15616478

[103] Thorning H Dixon L. Forty-five years later: the challenge of optimizing assertive community treatment. Curr Opin Psychiatry. (2020) 33(4):397–406. 10.1097/YCO.0000000000000615 32452942

[104] McHugo GJ Drake RE Teague GB Xie H. Fidelity to assertive community treatment and client outcomes in the new hampshire dual disorders study. Psychiatr Serv. (1999) 50(6):818–24. 10.1176/ps.50.6.818 10375153

[105] Aarons GA Hurlburt M Horwitz SM. Advancing a conceptual model of evidence-based practice implementation in public service sectors. Adm Policy Ment Health Ment Health Serv Res. (2011) 38(1):4. 10.1007/s10488-010-0327-7 PMC3025110 21197565

[106] Chinman M Imm P Wandersman A. Getting to Outcomes: Promoting Accountability Through Methods and Tools for Planning, Implementation, and Evaluation. Santa Monica, CA: RAND Corporation (2004).

[107] Hall G Hord SM. Change in Schools: Facilitating the Process. Albany, NY: SUNY Press (1987).

[108] Fixsen DL. Fidelity, not adaptation, is essential for implementation. Front Health Serv. (2025) 5:1–8. 10.3389/frhs.2025.1575179 PMC12061988 40353253

[109] Makary MA Daniel M. Medical error—the third leading cause of death in the US. Br Med J. (2016) 353:i2139. 10.1136/bmj.i2139 27143499

[110] Bedlington MM Braukmann CJ Kirigin KA Wolf MM, editors. Treatment Interactions, Delinquency, and Youth Satisfaction. San Francisco, CA: American Association of Behavior Therapy (1979).

[111] Bedlington MM Braukmann CJ Kirigin Ramp KA Wolf MM. A comparison of treatment environments in community-based group homes for adolescent offenders. Crim Justice Behav. (1988) 15:349–63. 10.1177/0093854888015003007

[112] Washington State Institute for Public Policy. Washington State’s Implementation of Functional Family Therapy for Juvenile Offenders: Preliminary Findings. Olympia, WA: Washington State Institute for Public Policy (2002) (Report No.: 02-08-1201).

[113] Alexander JF Parsons BV. Short-term family intervention: a therapy outcome study. J Consult Clin Psychol. (1973) 2:195–201. 10.1037/h0035181 4747932

[114] Alexander JF Pugh C Parsons BV Sexton TL. Functional family therapy. In: Elliott DS, editor. Book Three: Blueprints for Violence Prevention, 2nd ed. Golden, CO: Venture (2000). p. 3–79.

[115] Fixsen DL Baron RL Daly DL Tyler PM. Sustaining fidelity for 50 years: boys town and the teaching-family model. Resid Treat Child Youth. (2025) 42(4):518–41.

[116] Schoenwald SK Henggeler SW Brondino MJ Rowland MD. Multisystemic therapy: monitoring treatment fidelity. Fam Process. (2000) 39(1):83–103. 10.1111/j.1545-5300.2000.39109.x 10742933

[117] Tommeraas T Ogden T. Is there a scale-up penalty? Testing behavioral change in the scaling up of parent management training in Norway. Adm Policy Ment Health Ment Health Serv Res. (2017) 44:203–16. 10.1007/s10488-015-0712-3 PMC5306432 26715496

[118] Brunk M Chapman JE Schoenwald SK. Defining and evaluating fidelity at the program level: a preliminary investigation. Z Psychol. (2014) 222:22–9. 10.1027/2151-2604/a000162

[119] McIntosh K Mercer SH Nese RNT Ghemraoui A. Identifying and predicting distinct patterns of implementation in a school-wide behavior support framework. Prev Sci. (2016) 17(8):992–1001. 10.1007/s11121-016-0700-1 27549601

[120] Glisson C Schoenwald SK Hemmelgarn A Green P Dukes D Armstrong KS Randomized trial of MST and ARC in a two-level evidence-based treatment implementation strategy. J Consult Clin Psychol. (2010) 78(4):537–50. 10.1037/a0019160 20658810 PMC3951378

[121] Foege WH. House on Fire: The Fight to Eradicate Smallpox. Berkeley and Los Angeles, CA: University of California Press, Ltd. (2011).

[122] Ogden T Forgatch MS Askeland E Patterson GR Bullock BM. Large scale implementation of parent management training at the national level: the case of Norway. J Soc Work Pract. (2005) 19(3):317–29. 10.1080/02650530500291518

[123] Ogden T Bjørnebekk G Kjøbli J Patras J Christiansen T Taraldsen K Measurement of implementation components ten years after a nationwide introduction of empirically supported programs – a pilot study. Implement Sci. (2012) 7:49. 10.1186/1748-5908-7-49 22651221 PMC3405482

[124] Skogøy BE Maybery D Ruud T Sørgaard K Peck GC Kufås E Differences in implementation of family focused practice in hospitals: a cross-sectional study. Int J Ment Health Syst. (2018) 12(1):77. 10.1186/s13033-018-0256-5 30574174 PMC6299542

[125] Forgatch MS Patterson GR DeGarmo DS. Evaluating fidelity: predictive validity for a measure of competent adherence to the Oregon model of parent management training. Behav Ther. (2005) 36(1):3–13. 10.1016/S0005-7894(05)80049-8 16718302 PMC1464400

[126] Forgatch MS DeGarmo DS. Sustaining fidelity following the nationwide PMTO™ implementation in Norway. Prev Sci. (2011) 12(3):235–46. 10.1007/s11121-011-0225-6 21671090 PMC3153633

[127] Sigmarsdóttir M Forgatch M Vikar Guðmundsdóttir E Thorlacius Ö Thorn Svendsen G Tjaden J Implementing an evidence-based intervention for children in Europe: evaluating the full-transfer approach. J Clin Child Adolesc Psychol. (2018) 48:1–14. 10.1080/15374416.2018.1466305 29877721

[128] Gertner J. The Idea Factory: Bell Labs and the Great Age of American Innovation. NY: The Penguin Press (2012).

[129] Lewin K. Field Theory in Social Science. New York: Harper and Row (1951).

[130] Hussain ST Lei S Akram T Haider MJ Hussain SH Ali M. Kurt Lewin’s change model: a critical review of the role of leadership and employee involvement in organizational change. J Innov Knowl. (2018) 3(3):123–7. 10.1016/j.jik.2016.07.002

